# Analysis of Personality Traits in Patients with Hodgkin Lymphoma

**DOI:** 10.3390/jcm10081631

**Published:** 2021-04-12

**Authors:** Fatima Roso-Bas, Maria Dolores Alonso-Llobregat, Leyre Bento, Blanca Sanchez-Gonzalez, Ines Herraez, Pilar Garcia-Dilla, Catalina Vallespir, Francesca Rado, Raquel Rodriguez, Francesc Garcia-Pallarols, Irache Aguirre, Joan Bargay, Antonia Sampol, Antonio Salar, Antonio Gutierrez

**Affiliations:** 1 Clinical Practice and Biology of the Hematological Malignancies Research Group, IdISBa, Son Espases University Hospital, 07120 Palma de Mallorca, Spain; fatima.roso@ssib.es (F.R.-B.); mariad.alonsol@ssib.es (M.D.A.-L.); leyre.bento@ssib.es (L.B.); ines.herraez@hsll.es (I.H.); jbargay@hsll.es (J.B.); antonia.sampolm@ssib.es (A.S.); 2 Unit of Lymphoma, Department of Hematology, Son Espases University Hospital, 07120 Palma de Mallorca, Spain; 3 Department of Hematology, Hospital del Mar, 08003 Barcelona, Spain; 97894@parcdesalutmar.cat (B.S.-G.); mgarciad@psmar.cat (P.G.-D.); fgarcia1@imim.es (F.G.-P.); asalar@parcdesalutmar.cat (A.S.); 4 IMIM, Hospital del Mar Research Institute, 08003 Barcelona, Spain; 5 Department of Hematology. Son Llatzer University Hospital, 07120 Palma de Mallorca, Spain; 6 Service of Psychiatry, Son Espases University Hospital, 07120 Palma de Mallorca, Spain; catim.vallespir@gmail.com (C.V.); francesca.rado@ssib.es (F.R.); irache.aguirre@ssib.es (I.A.); 7 Psychosocial Support Team, Son Llatzer University Hospital, 07198 Palma de Mallorca, Spain; rrodriguezquintana@ohsjd.es

**Keywords:** five-factor model, personality traits, maladaptive traits, mental disorders, Hodgkin lymphoma, health outcomes, inflammatory biomarkers

## Abstract

Hodgkin lymphoma (HL) is a highly-curable malignancy mostly affecting young people. As far as we know, there is no published study that has analyzed personality profiles in HL nor their potential role in lymphomagenesis, natural history, or response to treatment. We aim to explore the personality traits of HL patients, as well as the prevalence of mental disorders and suicide ideas. We retrospectively identified all alive HL patients from three centers (Son Espases and Son Llatzer University Hospitals and Hospital del Mar of Barcelona) for using NEO Five-Factor Inventory (NEO-FFI) and Personality Inventory for DSM-5 Brief Form. Patients with HL showed significantly higher neuroticism scores and lower conscientiousness, extraversion, and openness. Considering maladaptive personality traits, HL patients showed higher levels of detachment and psychoticism. All of these translated into the fact that HL patients showed more than double the prevalence of mental illnesses (41%) and more than triple the prevalence of suicidal ideation or attempts than the general population (15 and 6%, respectively). An exploratory analysis of biomarkers associated with HL personality traits showed that higher scores of neuroticism correlated with more elevated erythrocyte sedimentation rate (ESR) and red cell distribution width (RDW), suggesting a potential link between neuroticism and proinflammatory activity in HL.

## 1. Introduction

Research on personality and its relationship with cancer from various approaches has a long history. Many studies have found personality to be a risk factor for the development of cancer, and other studies attribute to personality a prognostic value for response to treatment, in combination with other biological variables (i.e., immune response, neuroendocrine regulation), environmental (i.e., life stress), and behavioral (i.e., coping strategies, lifestyle). For a review, see [1,2]. Another relevant object of study is the impact of personality on the quality of life, together with other psychological variables, such as the dispositional level of optimism [3] or depression and anxiety [4]. These findings compose theories that establish the association between cancer and personality, however, much remains to be discovered regarding the specific nature of that link.

There are clinical suspicions in onco-hematology of differential personality characteristics in patients with Hodgkin lymphoma (HL) compared with patients with other neoplasms or the general population. Regarding literature about personality and HL, little research has been developed. One study, with a mixed sample of HL and non-Hodgkin lymphoma (NHL), found that scoring high in naiveté, conformity, and rigid adherence to social norms significantly shortened life years after treatment [5]. More recently, another study examined the association between personality traits and coping strategies with psychological distress in a sample of mixed lymphoma [6], concluding that coping strategies may partly explain the association between neuroticism (personality trait) and psychological stress.

The study of personality is complex and has generated many theories over time. We chose the comprehensive model called the “Big Five” or Five Factor Model (FFM) for our purposes. The FFM covers almost all the ways of explaining personality structure and concepts; it has a solid evidence base and has generated powerful measurement instruments [7,8,9]. From this perspective, personality is defined as a set of traits that explain thinking, feeling, and acting styles, and permit predicting the individual behavior [10]. These traits are grouped into five dimensions: neuroticism, extraversion, openness, to experience, agreeableness, and conscientiousness. In turn, each dimension is represented by six specific facets or micro-traits, which refer to thoughts, feelings, and acts. Individual behavior is the consequence of personality traits, social and cultural influences, which explain the behavioral variability in certain situations [11]. 

Personality is relatively stable throughout life; however, there is a maturational process where most changes reach the plateau over 30 years. Afterward, there are gradual and small changes across the life course [12,13,14]. New findings confirm that the pathological personality follows a similar evolution [15]. Given the parallelism and congruence between general and pathological personality, this study has focused on exploring these patients’ personalities from both perspectives.

Regarding the concept of the mental disorder, it is defined by Diagnostic and Statistical Manual of Mental Disorders, Fifth Edition. (DSM 5) [16] as a wide range of mental health conditions that temporally and substantially affect people’s ability to cope with daily life demands. Mental illnesses are associated with distress and problems functioning in social, work, or family activities. These include depression, bipolar affective disorder, schizophrenia, and other psychosis, dementia, intellectual disabilities, and developmental disorders, such as autism.

Under the FFM approach, several studies carried out in cancer patients conclude that neuroticism and conscientiousness are the most related traits to cancer [17,18]. Particularly, when comparing the profile between patients with cancer and without cancer, the cancer group present higher neuroticism and lower extraversion, agreeableness, and conscientiousness [19].

We aim to analyze these patients’ personality traits and other related psychological aspects, such as the prevalence of mental disorders and the ideas of suicide in our sample. Therefore, we hypothesize that HL patients may have different personality profiles compared to the general population.

## 2. Materials and Methods

### 2.1. Aims and Design of the Study

Our first objective is to identify the personality traits of HL patients through general and maladaptive personality traits. As a secondary objective, we aim to analyze whether these personality profiles differ from the general population’s personality and, in that case, to concretize these differences. Moreover, the prevalence of mental disorders (MD) in our sample will be found out. Finally, we aim to evaluate the relationship of these personality profiles with the cases’ clinical characteristics, potential laboratory biomarkers, such as erythrocyte sedimentation rate (ESR), reactive C-protein (RCP), or red cell distribution width (RDW) [20], and their potential impact on the clinical response to therapy and outcome.

This is a retrospective multicenter study performed in three hospitals: Son Espases University Hospital of Palma, Son Llatzer University Hospital of Palma, and Hospital del Mar of Barcelona. The study was approved by the Ethics Research Committee of the Balearic Islands (IB 3600/19, approval date 5-February-2018).

### 2.2. Sample and Procedure

To avoid selection bias, we identified all patients with HL diagnosis treated between January 2007 and December 2017 in all three hospitals from Pharmacy and Hematology Department databases. Eligibility criteria for this analysis included adult patients in the age range between 18 and 75 years. Exclusion criteria were language barrier and cognitive or neurological deterioration. 

Patients were contacted by phone and cited at the hospital. After being informed about the investigation and signing the consent, an interview was conducted, and the questionnaires were completed. The whole process lasted between 25 and 30 min. To reduce interferences, patients were asked to perform the interview and the questionnaires without their relatives’ presence.

### 2.3. Measures

#### 2.3.1. Interview

A semi-structured interview of 14 questions was conducted private setting to collect data about sociodemographic variables, substance use, stressful events in life, coping, psychological disorders, suicidal ideation, and attempts. Data about psychological background were verified by reviewing medical records.

#### 2.3.2. NEO Five-Factor Inventory (NEO-FFI)

The NEO-FFI is a 60-item instrument developed within the Five Factor Model (FFM) framework by Costa and McCrae and adapted to the Spanish population [21]. A five-point agreement scale was used, ranging from 1 (strongly disagree) to 5 (strongly agree). Overall, general personality can be structured in 5 traits: neuroticism (i.e., the tendency to experience negative emotions such as anxiety and depression); extraversion (i.e., the tendency to be friendly, assertive, warm, cheerful, and active); openness to experience (i.e., the trend to be creative, unconventional, emotionally and artistically sensitive); agreeableness (i.e., the tendency to be trustworthy, modest, altruist, and collaborative); and conscientiousness (i.e., a tendency to be persistent, organized, reliable, and strict with rules).

#### 2.3.3. Personality Inventory for DSM-5 Brief Form (PID-5-BF)

This questionnaire is a 25-item instrument developed by Krueger [22]. The questionnaire has been validated in Spain [23], while the brief version has yet to validate. The PID-5-BF evaluates personality disorders proposed in Section III of the DSM-5. This measure asks participants to rate statements on a 4-point Likert-type scale from 0 (very false or often false) to 3 (very true or often true). Scores are calculated by averaging items, as indicated in the development study.

From this perspective, personality disorders are also dimensioned in 5 traits: negative affectivity (i.e., the tendency to experience negative emotions), detachment (i.e., characterized by social isolation, introversion, and anhedonia), antagonism (i.e., aggressive tendencies with assertions of dominance and grandiosity), disinhibition (i.e., impulsivity and sensation-seeking), and psychoticism (i.e., a disconnection from reality and a tendency to experience illogical thought patterns).

### 2.4. Statistical Analysis

Variables following binomial distributions were expressed as frequencies and percentages. Comparisons between qualitative variables were made using the Fisher’s exact or Chi-square tests. Comparisons between quantitative and qualitative variables were performed through non-parametric tests (Mann–Whitney U or Kruskal–Wallis).

Time to event variables were measured from the date of therapy onset and were estimated according to the Kaplan–Meier method. Comparisons between the variables of interest were performed by the log-rank test. All *p*-values reported were 2-sided, and statistical significance was defined at *p* < 0.05.

The personality scores obtained in our sample were compared with the national reference samples. In the case of general personality traits, the one-sample t-test was applied since they followed normal distributions. Regarding the measures of pathological features, some deviated from normality, the one-sample Wilcoxon signed-rank test was applied to contrast the median of our sample with the median of the reference population.

## 3. Results

### 3.1. Characteristics of the Patients

We included 95 patients diagnosed with HL and treated between January 2007 and December 2017. Patient and sociodemographic characteristics are summarized in Table 1 and Table 2. Briefly, the median age was 37 years, and most patients had an early stage (59%). 59% of early stage patients and 24% of advanced cases had high-risk scores, German Hodgkin Study Group (GHSG) criteria ≥ 1 or international prognostic score (IPS) > 3, respectively. Most patients received adriamycin, bleomycin, vinblastine, and dacarbazine (ABVD) (94%) as frontline therapy, and 85% obtained a complete response (CR). With a median follow-up of 72 months, 6-year event-free survival was 79% (Table 1). In regard to sociodemographic characteristics, most patients were single (48%), with high-level education, without habits of substance abuse (55%), but reporting moderate or intense vital stressors (66%), including loss of work, death of relatives or friends, and sentimental issues.

### 3.2. General Personality Traits

**Figure 1:**
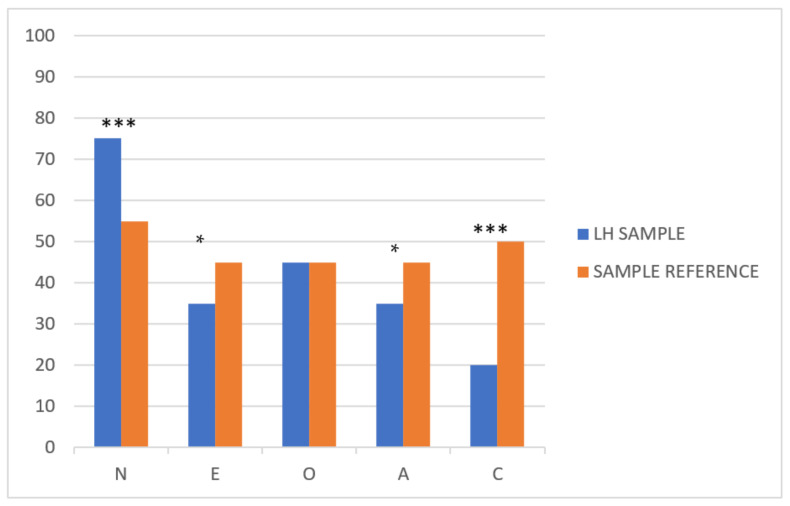
Profile of general personality traits in Hodgkin lymphoma (HL) and reference sample in percentiles. Levels of significance: *: <0.05; ***: <0.001.

We first calculated the mean of each personality trait for the whole of our sample to establish the personality profile. Then, these averages were compared with the mean reference. As shown in Table 3, except for openness, all the traits were statistically significant. To illustrate the profile, the mean of the scores has been transformed into T scores (M = 50, SD = 10) to easily compare the profile of patients with HL with the values of the sample reference (see Figure 1).

Comparing the NEO-FFI scores obtained in our sample with the scale of the Spanish population [21], neuroticism is close to the 75th percentile, extraversion in the 35th percentile, openness in the 50th percentile, agreeableness in the 40th percentile, and conscientiousness in the 20th percentile (see Figure 1). So, although the four traits (neuroticism, extraversion, agreeableness, and conscientiousness) were significantly different compared with the general population, the main differences were observed in both neuroticism and conscientiousness.

### 3.3. Maladaptive Personality Traits

Table 4 shows the average scores for each trait. A non-parametric contrast was performed comparing the sample medians with the reference ones, because only negative affectivity adjusted to normality (Kolmogorov-Smirnov (K-S) = 1.80). Negative affectivity, detachment, and psychoticism scored significantly higher than reference scores. In this case, the main differences were observed in negative affectivity and psychoticism, which were significantly higher in HL patients.

### 3.4. Prevalence of Mental Disorders Others Related Psychological Aspect

In our study, up to 39 (41%) of the patients had been diagnosed with a mental disorder before HL diagnosis. Mood and anxiety disorders were the most frequent diagnoses in our sample (*n* = 33) (35%). As for suicide, six patients (6%) presented suicide attempts at some point in their lives, and 14 patients (15%) had suicidal thoughts. Overall, 35 patients (37%) have needed psychopharmacologic therapy throughout their lives, and 41 patients (43%) declared psychological assistance. These results are presented in Table 5 and compared with general population data.

### 3.5. Exploratory Analysis of the Relationship of Personality Traits and Clinical Characteristics of HL and Potential Biomarkers

As neuroticism and conscientiousness were the two personality traits with more significant differences between HL patients and the general population, we tested the relationship between standard clinical parameters, potential laboratory biomarkers in HL, and these personality traits. The most prominent findings were obtained with neuroticism. Higher neuroticism scores were significantly related to the female gender and HL patients with previous history of mental illness, requiring mental health assistance, or prior autolytic ideation/behavior. Considering potential biomarkers, we found that higher neuroticism scores were related to higher ESR or RDW levels. Moreover, 26% of HL patients with elevated ESR levels showed very high neuroticism levels (>27), while only 9% of those patients with normal ESR (*p* = 0.046). Similarly, patients with high RDW had significantly more cases with very high neuroticism when compared to patients with normal RDW (29% vs. 7%; *p* = 0.023). RCP levels also tended to be associated with a higher proportion of patients with high levels of neuroticism: 23% vs. 9% in the case of normal RCP. Considering conscientiousness, we did not find significant relationships with any clinical characteristic or biomarker. However, there was a trend to a higher number of patients with very low responsibility in younger patients (<45 years), IPS > 3, and the presence of B-symptoms (Table 6).

There was no relation between response to first line therapy or event-free survival (EFS) and neuroticism or conscientiousness. However, we observed a non-significant tendency to a worse 6y-EFS associated to very high neuroticism (65%) versus 81% in patients with lower neuroticism scores (*p* = 0.46). 

## 4. Discussion

To our knowledge, this is the first report of specific personality traits related to HL. We found that patients with HL have significantly higher neuroticism levels, lower levels of responsibility, and a higher prevalence of mental illnesses compared to the general population. Personal profiles are relatively consistent and stable all life [12], so it is not easy to ascertain the role of these psychologic traits in this type of lymphoma. However, one could discard that they are the consequence of this malignancy. We should try to discover if these personality traits could have any relationship in their etiology or pathogeny, together with other genetic and microenvironmental causes.

Our research results configure a characteristic personality pattern in which high neuroticism and low consciousness are highlighted, which is consistent with many other investigations that study personality in cancer patients [17,18]. The profile of high neuroticism and low conscientiousness correlates with many adverse health outcomes in the general population [30,31,32,33]. Interestingly, in oncology patients receiving chemotherapy, it has been confirmed a personality style configured by these two traits called “distressed personality class” that has been associated with more intense levels of anxiety, depression, cancer symptoms [34], and a less adaptive repertoire of coping strategies [35].

When scores on both traits are reversed, a protection profile is established. Thus, low neuroticism and mostly high conscientiousness can be considered as a protective factor of health and, in general, to the best physical health [36]. This is partly explained by the manifestation of healthy behaviors as physical activity [37] or test for cancer early detection [18], among others. Moreover, people who are emotionally stable and conscientious tend to live longer [38,39].

Research on maladaptive personality traits and health is much scarcer than that found on basic traits. However, longitudinal studies conclude that measures of maladaptive traits account for more variance than measures of general personality traits. Specifically related to cancer, the percentage of variance explained by maladaptive variants was found to be substantially higher than that defined by general traits (4% to 1%, respectively) [40]. 

Within the FFM framework, the correspondences between general and pathological traits are as follows: negative affectivity with neuroticism, detachment with low extraversion, antagonism with low agreeableness, disinhibition with low conscientiousness, and psychoticism with high openness. As expected and consistent with the result obtained in the neuroticism trait, patients with HL in our sample scored high in negative affectivity. On the other hand, psychoticism has received the highest score, in contrast with openness, which did not differ from the population mean. Other studies have detected this inconsistency; thus, it seems that the relationship between openness and psychoticism is not fully clarified [41,42]. Nevertheless, the most relevant result is the psychoticism score in our sample, which is more than double the one obtained in the general population. These two maladaptive traits can shape a personality profile whereby the person tends to experience negative feelings, restricted affectivity, unusual perceptions, and thoughts, and exhibits eccentric behavior. 

The frequency of diagnosis of mental disorders in our sample was 41%, which is surprisingly high considering that the percentage of mental health diagnosis in Spain is 15.4% (Balearic Islands: 11.4%; Catalonia: 15%) [24]. This difference is slightly less than the 25,9% obtained in the European Study of the Epidemiology of Mental Disorders (ESEMeD) [43]. Regarding mood disorders, the prevalence of global anxiety (28%) and depression (21%) was also higher in our sample compared with the general Spanish population, which is 6,7% for both mood disorders (anxiety [25] and depression [26,27]). Moreover, these differences are very significant when the differential distribution of genders is considered (Table 5).

Of our sample, 6% presented suicide attempts at some point in their lives, and 15% suicide thoughts. Suicide and self-inflicted injuries continue to be the leading cause of unnatural death in Spain (2018), with 3539 deaths (7.25 per 100,000 inhabitants), representing the death of 10 people a day; 3 out of 4 are men (2619) and 25% women (920). In 2018, the Balearic Islands would be in the fourteenth position (ratio of 6.08 per 100,000 inhabitants) behind Catalonia (rate of 6.75 per 100,000 inhabitants) [28,29,44].

We could not find significant relationships between personality traits and most common prognostic factors in HL or progression-free survival. However, as HL has a well-known inflammatory background, where dysregulation of cytokines and microenvironment have an important pathogenic role, we also decide to test several pro-inflammatory biomarkers, such as ESR, RCP, or RDW. In this analysis, the most remarkable results were obtained with neuroticism, as patients with high ESR and RDW were significantly associated with a higher proportion of patients showing very high scores in the neuroticism trait. ESR is a well-known adverse prognostic factor in HL and is directly linked to the patient’s pro-inflammatory status [45]. RDW is associated with age, comorbidities, and systemic inflammation [46,47]. All of this could suggest a potential link among neuroticism, low conscientiousness, pro-inflammation, and HL. High neuroticism and low conscientiousness are frequently implicated in health-risk behaviors, and previous reports have found a relationship between elevated RCP, increased interleukin 6 (IL-6), and these psychological traits [48]. Additionally, neuroticism has been linked to higher oxidative stress [49]. Future research will have to study the precise etiopathogenic mechanisms implicated in these findings and the necessary implication of different genetic predisposition.

We could not find a significant relationship between any of the personality traits and the outcome. However, we observed a non-significant tendency to a worse 6 y-EFS associated with very high Neuroticism: 65% versus 81% in patients with lower neuroticism scores. Although this tendency has also been reported in other malignancies [50], and should be confirmed in a larger series of HL patients. The particular knowledge of the role of these traits in HL could be associated with the etiology and pathogeny of HL, having implications in the follow-up of the vast number of survivors after therapy.

There are several limitations associated with this research study. First, the results depend on the answer to the self-report instruments; individuals can over- or underreport what is known as test response bias. This can be due to socially desirable responses [51], and many authors suggest that it is necessary to develop validity scales to screen this bias for assessing maladaptive personality traits [52,53]. Yet, in personality psychology, the preferred method is to ask people to answer questions about their way of being and behaving [54]. Regarding measures of general traits, the agreement between various sources on an individual’s position in the five dimensions is substantial [10]. In our work, to minimize this possible bias, the data were collected by researchers who had no clinical relationship with the patients; they insisted on the importance of honest answers, and the confidentiality of the collected data were guaranteed. Another limitation in our study is related to the reduced versions of the questionnaires that have been used; this implies that we have explored personality traits at a general level. Therefore, we cannot examine personality facets, which are the basic elements that specify personality. Moreover, the study’s retrospective nature reduces the value of the conclusions regarding the impact of personality traits in survival. To overcome this potential bias, we included all alive patients in our study, and currently, we are working on a prospective project inside the Spanish Group of Lymphoma (GEL/TAMO). Finally, our manuscript describes the presence of a differential profile of personality traits and prevalence of mental discomfort in HL compared to the general population. However, future works, including patients of other lymphomas or malignancies, should investigate if the observed differences are specific to HL, or just a general finding in cancer. Again, currently, we are working in a prospective project, including several types of lymphoma, other than HL.

## 5. Conclusions

1. To our knowledge, we present, for the first time, the presence of a differential profile of personality traits in HL when compared to the general population.
2. Patients with HL showed significantly higher scores of neuroticism and lower conscientiousness, extraversion, and openness.
3. Considering maladaptive personality traits, HL patients showed higher levels of detachment and psychoticism.
4. All of these translated into the fact that HL patients show a higher prevalence (more than double) of mental illnesses and suicidal ideation or attempts (more than triple) than the general population.
5. An exploratory analysis of biomarkers associated with HL personality traits showed that higher scores of neuroticism correlated with higher levels of ESR and RDW, which suggests a potential link between neuroticism and pro-inflammatory activity in HL. Further studies should confirm this observation.

## Figures and Tables

**Table 1:** Patient characteristics.

Clinical Characteristics	*n* (%)
Median age (range) (years)	37 (14–76)
Age > 45 years	35 (37%)
Age > 60 years	12 (13%)
Sex (Male/Female)	49 (52%)/46 (48%)
Medical center: - HUSE - HSLL - HMAR	60 (63%) 20 (21%) 15 (16%)
AA stage: - Early - Advanced	55 (59%) 38 (41%)
B-symptoms:	33 (36%)
Bulky disease:	20 (22%)
ECOG PS >1:	7 (8%)
GHSG ≥1 (Early HL):	23 (59%)
IPS > 3 (Advanced HL):	8 (24%)
Frontline Chemotherapy: - ABVD - BV-ABD - MOPP - Other	89 (94%) 2 (2%) 1 (1%) 2 (2%)
Radiotherapy:	29 (31%)
Response: - CR - PR - SD/PD	81 (85%) 5 (5%) 9 (9%)
Median follow-up (range) (months)	72 (5–415)
6 years-EFS (95%CI)	79% (74–84)
Progression	17 (18%)
Exitus	1 (10%)

HUSE: University Hospital Son Espases; HSLL: Hospital Son Llatzer; HMAR: Hospital del Mar Barcelona; AA: Ann Arbor; ECOG PS: Eastern Cooperative Oncology Group Performance Status; ABVD: adriamycin, bleomycin, vinblastine, and dacarbazine; BV-ABD: brentuximab vedotin, adriamycin, vinblastine and dacarbazine; MOPP: mechlorethamine, vincristine, procarbazine, and prednisone; CR: complete response; PR: partial response; SD/PD: stable disease/progression disease; EFS: event-free survival.

**Table 2:** Sociodemographic characteristics.

	*n* (%)
Civil Status	
Single	46 (48.4)
Married	38 (40)
Separated/Divorced	8 (8.4)
Widowed	3 (3.2)
Educational level	
Uneducated	2 (2.1)
Primary	18 (18.9)
Secondary	31 (32.6)
High School	42 (44.2)
Substance abuse	
No habits of abuse	52 (54.7)
Tobacco	13 (13.7)
Alcohol	7 (7.4)
Cannabis	5 (5.3)
Several substances	18 (18.9)
Vital stressor	
No vital stressor	28 (29.5)
Mild	3 (3.2)
Moderate	10 (10.5)
Intense	53 (55.8)

**Table 3:** Scores on general personality traits.

NEO-FFI	α	Reference Population *		HL Sample		*t* **		
		Mean	SD	Mean	SD	Mean Difference	CI (95%)	*p*
Neuroticism	0.852	15.35	7.40	20.61	9.31	5.26	(3.30; 7.23)	**0.000**
Extraversion	0.829	32.59	6.35	30.36	7.94	−2.22	(−3.93, −0.52)	**0.011**
Openness	0.774	28.64	6.56	28.64	7.69	0.006	(−1.68, 1.69)	0.994
Agreeableness	0.676	32.79	5.67	31.36	6.35	−1.42	(−2.77, −0.067)	**0.040**
Conscientiousness	0.798	36.01	6.02	32.21	7.35	−3.79	(−5.33, −2.25)	**0.000**

* NEO Five-Factor Inventory (NEO-FFI) (TEA editions scale, [21]), ** One-sample *t*-test. Bold: statistically significant *p*-value.

**Table 4:** Scores on maladaptive personality traits.

PID-5-BF	α	Reference Population *			HL Sample			Z **
		M	SD	Med	M	SD	Med	*p*
Negative affectivity	0.720	0.940	0.431	0.906	1.384	0.704	**1.40**	**0.000**
Detachment	0.710	0.626	0.432	0.562	0.758	0.620	**0.70**	**0.006**
Psychoticism	0.681	0.392	0.407	0.250	0.821	0.608	**0.80**	**0.000**
Antagonism	0.603	0.497	0.419	0.400	0.302	0.372	**0.20**	**0.002**
Disinhibition	0.752	0.742	0.465	0.687	0.780	0.638	0.60	0.224

PID-5-BF: Personality Inventory for DSM-5 Brief Form; HL: Hodgkin lymphoma * Ruiz et al. (2019), [15] ** Wilcoxon signed rank test for one sample. Bold: statistically significant *p*-value.

**Table 5:** Prevalence of mental disorders in our series compared with general population data.

	Current Series			Other Series		
	Global	Male	Female	Global	Male	Female
Mental disorders (%)	39 (41%)	18 (37%)	21 (46%)	15.4% [24]	9.7% [24].	20.2% [24].
Mood disorders: - Anxiety - Depression	33 (35%) 26 (28%) 20 (21%)	8 (16%) 7 (14%)	19 (41%) 12 (26%)	6.7% [25] 6.7%) [26]	4.3% [25] 4% [27]	9.1% [25] 9% [27]
Suicidal ideation	14 (15%)	6 (12%)	8 (17%)	4% [28]	-	-
Suicidal attempts	6 (6%)	4 (8%)	2 (4%)	1.48% [29]	-	-

-: not available.

**Table 6:** Personality traits and clinical characteristics/biomarkers in HL.

Characteristics/Biomarkers	High Neuroticism (>27). *n* (%)	*p*	Very Low Conscientiousness (<26). *n* (%)	*p*
Age: - <45 - ≥45	13 (23%) 5 (15%)	0.42	13 (22%) 3 (9%)	0.12
Sex: - Male - Female	4 (9%) 14 (32%)	0.009	7 (15%) 9 (20%)	0.52
AA stage: - I–II - III–IV	10 (19%) 7 (20%)	1	10 (19%) 5 (14%)	0.58
B-symptoms: - Yes - No	7 (22%) 9 (17%)	0.58	8 (26%) 7 (13%)	0.12
Bulky mass: - Yes - No	6 (30%) 10 (16%)	0.15	3 (15%) 12 (19%)	0.7
IPS: - 0–3 - >3	8 (14%) 3 (30%)	0.23	7 (13%) 3 (30%)	0.16
ESR: - Normal - High	3 (9%) 11 (26%)	0.046	6 (17%) 7 (17%)	0.96
RCP: - Normal - High	2 (9%) 10 (23%)	0.2	5 (23%) 6 (14%)	0.37
RDW: - Normal - High	2 (7%) 9 (29%)	0.023	4 (13%) 7 (23%)	0.29

AA: Ann Arbor; IPS: international prognostic score; EST: erythrocyte sedimentation rate; RCP: reactive C-protein; RDW: red blood cell distribution width.

## Data Availability

The data presented in this study are available on request from the corresponding author. The data are not publicly available due to privacy restrictions.
